# Arteriovenous Malformation of the Ureter: A Rare Urological Entity Managed With Endoscopic Laser Ablation

**DOI:** 10.1155/criu/3037053

**Published:** 2026-04-15

**Authors:** A. Anber, J. Kyeremeh, A. Coscione, L. Dragos, A. Ahmed

**Affiliations:** ^1^ Department of Urology, Addenbrookes Treatment Centre, Cambridge University Hospitals NHS Foundation Trust, Cambridge, UK, cuh.org.uk; ^2^ Department of Surgery, University of Cambridge, Cambridge, UK, cam.ac.uk

**Keywords:** endoscopic resection, flexible ureteroscopy, haematuria, laser ablation, rare urological lesions, ureteric arteriovenous malformation

## Abstract

**Background:**

Arteriovenous malformations (AVMs) of the urinary tract are exceedingly rare, especially when arising from the ureter. Presenting often with haematuria and obstructive symptoms, these lesions pose diagnostic and therapeutic challenges, with no established management guidelines.

**Case Presentation:**

We report a case of a 41‐year‐old woman who presented with left‐sided flank pain, fever and visible haematuria. Initial imaging revealed hydronephrosis and a proximal ureteric soft tissue lesion. Flexible ureteroscopy identified a large, pale and fleshy intraluminal mass extending into the pelviureteric junction. Laser ablation and endoscopic resection were performed, and histopathology confirmed a polypoid AVM. Postoperative recovery was uneventful, apart from a conservatively managed renal abscess. The patient remained asymptomatic at 12‐month follow‐up.

**Conclusion:**

This case highlights a rare presentation of ureteric AVM and supports flexible ureteroscopy with laser ablation as a viable and minimally invasive treatment modality. Clinicians should consider vascular anomalies in patients with unexplained haematuria, particularly when conventional aetiologies are excluded.

## 1. Introduction

Haematuria is a common urological symptom affecting individuals across all age groups. It is broadly categorised into visible (macroscopic) and nonvisible (microscopic) haematuria, each potentially indicating serious underlying pathology. Common causes include urinary tract infections, nephrolithiasis, malignancies and benign prostatic hyperplasia [1]. Despite the broad differential for haematuria, vascular anomalies such as AVMs are often overlooked. AVMs of the urinary tract are exceptionally rare, particularly those originating from the ureter [2, 3]. Fewer than 10 such cases have been reported in the English literature [4]. When present, AVMs may manifest with painless haematuria, flank pain or obstructive symptoms [3]. No established guidelines currently exist for their management. We present a rare case of ureteric AVM successfully managed with laser ablation and resection, adding to the limited number of documented instances of this treatment approach.

Timely recognition of such rare entities is essential to prevent recurrent symptoms and complications.

## 2. Case Presentation

A 41‐year‐old woman presented to the emergency department with left‐sided flank pain, fever, nausea, vomiting and visible haematuria with clots. These symptoms developed 1 day after being treated by her general practitioner for a presumed urinary tract infection with oral nitrofurantoin. She had a history of recurrent urinary tract infections since childhood, managed empirically with antibiotics.

Her past medical history included sciatica due to L5/S1 disc herniation, treated with a left S1 CT‐guided nerve root injection. She was taking regular analgesia (naproxen and co‐codamol). Surgical history included a laparoscopic cholecystectomy. There was no history of urolithiasis, prior pelvic surgeries or pelvic radiation exposure. Smoking was stopped 10 years ago and had no known drug allergies.

On examination, she was in acute distress. Her blood pressure was 154/90 mmHg, heart rate 100 bpm and pain score 7/10, with otherwise normal vital signs. Significant left costovertebral angle tenderness was noted. No palpable abdominal masses were detected. Laboratory findings included elevated creatinine (92 *μ*mol/L), WCC of 13.9 × 10^9^/L and CRP of 33 mg/L.

**Figure 1:**
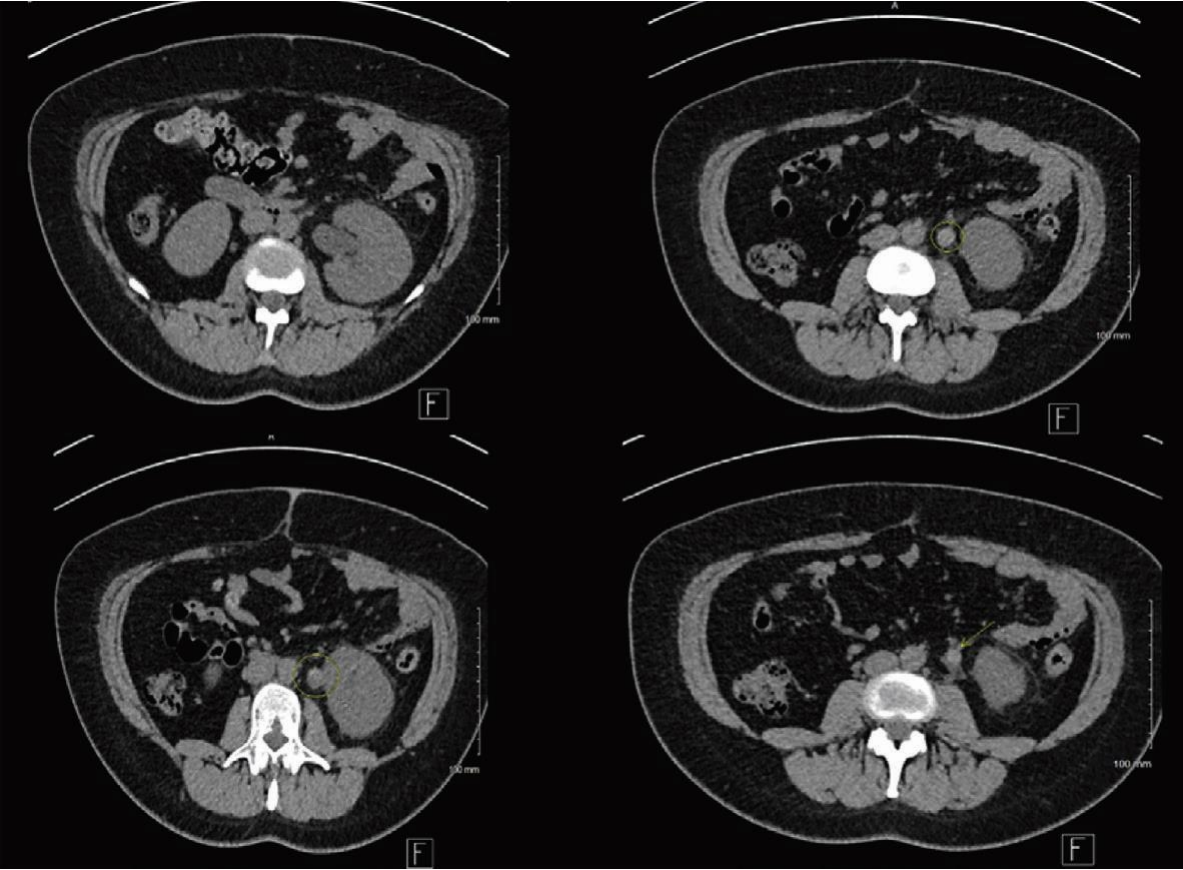
Noncontrast CT abdomen and pelvis showing left hydroureter and hydronephrosis in addition to an ipsilateral lesion of the proximal ureter.

A noncontrast CT scan (seen in Figure 1) of the abdomen and pelvis was initially requested to exclude acute renal colic secondary to obstructive stone disease, which revealed left‐sided hydronephrosis and hydroureter with perinephric fat stranding. A soft tissue density (HU 65) was noted in the proximal left ureter, without obvious radio‐dense calculi.

**Figure 2:**
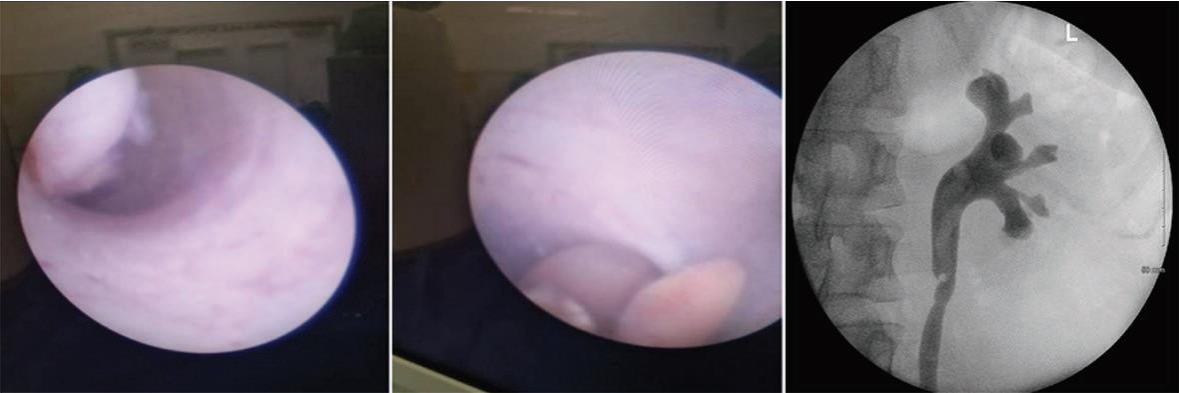
Gross appearance of the AVM tissue extending to the left PUJ and the retrograde pyelogram showing a well‐defined filling defect.

The patient was admitted and treated with intravenous fluids and antibiotics. She responded well and was discharged after 2 days. An urgent outpatient ureteroscopy with possible biopsy was scheduled. Three weeks later, elective left ureteroscopy was performed. Cystoscopy showed a normal bladder. Ureteroscopy identified a large, pale and fleshy mass extending from the proximal ureter into the pelviureteric junction (PUJ) and collecting system. No malignant‐appearing lesions were seen. Urine cytology was obtained. A retrograde pyelogram shown in Figure 2 revealed a well‐defined filling defect.

**Figure 3:**
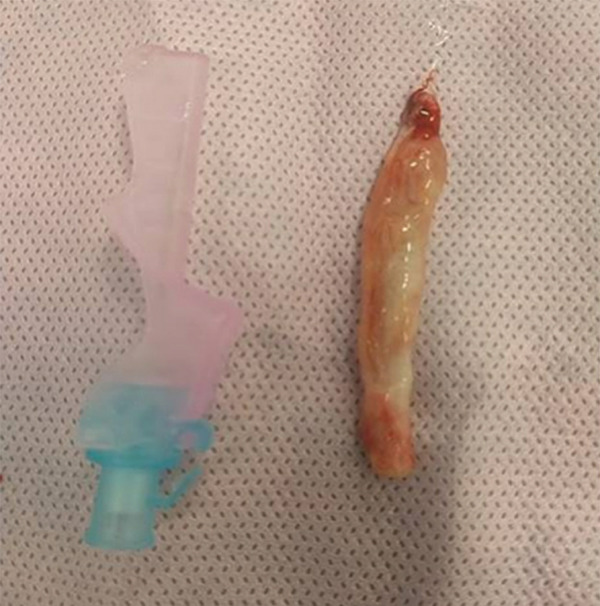
Gross image of the excised polyp following laser ablation.

A Boston Scientific LithoVue single‐use 7.7‐Fr flexible ureteroscope was advanced through the ureter to the lesion without an access sheath. Laser ablation was then performed using a holmium: YAG laser (1 J, 10 Hz) with a 200‐*μ*m fibre. A zero‐tip basket was used to extract the lesion (Figure 3), and the specimen was obtained and appropriately labelled. Postprocedural inspection demonstrated a smooth ureteric wall with no residual lesion, perforation or injury. No abnormality was detected in the renal pelvis. A JJ ureteric stent was placed for 2 weeks and subsequently removed.

**Figure 4:**
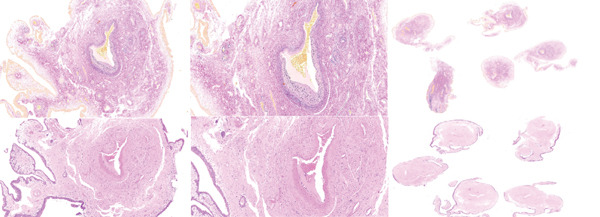
Urine cytology showing pseudopapillary groups of urothelial cells present with no high‐grade urothelial cancer cells.

Histopathology revealed a polypoidal mucosal lesion with ill‐defined proliferation of variably sized blood vessels, some with thick hyaline walls. Internal elastic lamina and muscular tunica media were variably present. Capillaries without muscular or elastic components were noted. The surface urothelium was unremarkable. These findings confirmed an arteriovenous malformation (seen in Figure 4). Urine cytology showed pseudopapillary groups of urothelial cells present with no high‐grade urothelial cancer cells.

One week later, the patient returned with flank pain and fever. Imaging confirmed appropriate stent placement, but contrast‐enhanced CT showed a likely evolving left upper pole renal abscess. This was managed conservatively with antibiotics, and the patient improved. She was discharged with an 8‐week follow‐up and ultrasound. At follow‐up, ultrasound showed no abnormalities. The patient remained asymptomatic with no recurrence of haematuria or infections over a 12‐month period.

## 3. Discussion

Ureteric AVMs are extremely rare vascular malformations, with very few cases described in literature. First reported by Kaplan et al. [3] in 1992, subsequent cases by Yadunandan et al. [4] and Calabrese et al. [5], have illustrated variable presentations, most commonly haematuria and obstructive symptoms. In contrast to more commonly encountered AVMs in the renal parenchyma or pelvis, ureteric AVMs are anatomically constrained and may be misinterpreted as fibroepithelial polyps, transitional cell carcinoma or inflammatory lesions on imaging and endoscopy [6]. This diagnostic ambiguity poses a risk of both undertreatment and overtreatment. For instance, diagnostic uncertainty may lead to overtreatment such as segmental ureterectomy or nephroureterectomy.

Contrast‐enhanced CT may demonstrate enhancement and should be considered, particularly when a vascular lesion is suspected [7].

Our case is notable for several reasons. Firstly, it reinforces the importance of considering rare vascular anomalies when evaluating persistent haematuria, especially in patients with a long‐standing history of urinary tract infections but no evidence of calculi or malignancy. Although imaging (such as CT or MRI) may suggest a soft tissue lesion, only endoscopic evaluation and biopsy can definitively identify an AVM. In this case, the lesion appeared polypoid and pale but not overtly malignant, highlighting the limitations of visual inspection alone.

Brush cytology may be helpful when malignancy cannot be excluded; however, it is not routinely used in suspected vascular lesions. Endoscopic visualization and biopsy remain crucial for definitive diagnosis.

Secondly, the case underscores the efficacy and safety of flexible ureteroscopy with laser ablation as a first‐line intervention. The use of a 200‐micron holmium laser fibre allowed for precise, controlled ablation of the vascular lesion without causing ureteric perforation or stricture. Traditional management approaches for ureteric AVMs have included open or laparoscopic resection, often with segmental ureterectomy or nephroureterectomy when malignancy could not be excluded [8]. The minimally invasive approach used here allowed for both therapeutic excision and histological diagnosis in a single procedure, reducing patient morbidity and shortening hospital stay.

Thirdly, this case contributes to the limited global literature on ureteric AVMs. According to a 2021 literature review by Yadunandan et al., only five cases had been reported in the English‐language literature [4]. The consistent themes amongst these reports are the absence of specific imaging findings, frequent misdiagnosis and the ultimate need for histopathological confirmation. This case adds to the limited number of documented instances where a ureteric AVM was successfully treated using laser ablation.

Furthermore, this case illustrates a postoperative complication of renal abscess formation following stent placement, which was managed successfully without surgical intervention. This emphasizes the importance of close postprocedural monitoring in such vascular lesions, particularly when tissue handling may lead to microinjury or transient ischaemia [9].

Finally, the report adds value by proposing a feasible diagnostic and therapeutic pathway for similar cases: When a filling defect is detected without a clear aetiology and malignancy is not overtly suspected, ureteroscopic visualization and laser ablation with biopsy offer a diagnostic and potentially curative intervention [5, 10]. As more cases are reported, a standardized approach to managing ureteric AVMs may emerge, potentially integrating newer imaging modalities like contrast‐enhanced ultrasound or digital ureteroscopes with narrow‐band imaging to enhance vascular pattern recognition.

## 4. Conclusion

This case reinforces the importance of considering vascular malformations in the differential diagnosis of unexplained haematuria, particularly when initial imaging reveals a nonspecific ureteric filling defect. A structured diagnostic pathway, including contrast‐enhanced imaging, retrograde pyelography and endoscopic evaluation, can help distinguish AVMs from mimicking entities such as urothelial tumours or fibroepithelial polyps. Flexible ureteroscopy with laser ablation provides a safe, minimally invasive and organ‐preserving option for both diagnosis and definitive treatment. As additional cases are reported, they will contribute to the development of clearer diagnostic criteria and management guidelines for this rare urological entity.

## Funding

No funding was received for this manuscript.

## Consent

Written informed consent was obtained from the patient for publication of this case report and any accompanying images.

## Conflicts of Interest

The authors declare no conflicts of interest.

## Data Availability

Data sharing is not applicable to this article as no datasets were generated or analysed during the current study.

## References

[1] King K. and Steggall M. , Haematuria: From Identification to Treatment, British Journal of Nursing. (2014) no. 23Supplement 9, S28–S32, 10.12968/bjon.2014.23.Sup9.S28, 2-s2.0-84902082949, 24820511. 24820511

[2] Singh K. J. , Raghvendran M. , Suri A. , and Mandhani A. , Renal and Ureteral Arteriovenous Malformation, Indian Journal of Urology. (2004) 20, no. 2, 69–70, 10.4103/0970-1591.37181.

[3] Kaplan S. A. , Brown W. , Bixon R. , O′Toole K. , and Benson M. C. , Arteriovenous Malformation of Ureter, Urology. (1992) 40, no. 5, 450–452, 10.1016/0090-4295(92)90462-6, 2-s2.0-0026451848. 1441045

[4] Yadunandan A. G. , Pai N. , Hegde S. , and Rajeev T. P. , Arteriovenous Malformation of Ureter Presenting Without Hematuria: An Unusual Presentation of a Rare Disease, Urology Annals. (2021) 13, no. 2, 177–179, 10.4103/UA.UA_24_20, 34194147. 34194147 PMC8210726

[5] Calabrese E. , Baghdanian A. H. , Zagoria R. J. , and Behr S. C. , Arteriovenous Malformation of the Ureter Diagnosed by CT Urogram, Urology Case Reports. (2018) 19, 20–22, 10.1016/j.eucr.2018.04.001, 2-s2.0-85045615959, 29888180. 29888180 PMC5991259

[6] Tang C. N. , Law I. C. , Iu P. P. , and Yip A. W. C. , Arteriovenous Malformation of the Ureter–a Rare Cause of Haematuria, British Journal of Urology. (1997) 80, no. 3, 500–501, 10.1046/j.1464-410X.1997.00384.x, 9313681. 9313681

[7] Sech S. M. , Saboorian M. H. , Ashfaq R. , Somerville P. J. , and Pearle M. S. , Polypoid Arteriovenous Malformation of the Ureter, Journal of Urology. (1997) 158, 1903–1904, 10.1016/S0022-5347(01)64166-1, 2-s2.0-0030879661, 9334628. 9334628

[8] Liu F. , Guo W. , Zhou X. , Ding Y. , Ma Y. , Hou Y. , Kong X. , and Wang Z. , Laparoscopic Versus Open Nephroureterectomy for Upper Urinary Tract Urothelial Carcinoma: A Systematic Review and Meta-Analysis, Medicine. (2018) 97, no. 35, e11954, 10.1097/MD.0000000000011954, 2-s2.0-85052927904, 30170392. 30170392 PMC6393120

[9] Li K. , Wang J. , and Sessler D. I. , Continuous Ward Monitoring and Intensive Postoperative Management, Chinese Medical Journal. (2024) 137, no. 6, 631–632, 10.1097/CM9.0000000000002997, 38384165. 38384165 PMC10950178

[10] Ten Donkelaar C. S. , Houwert A. C. , Ten Kate F. J. W. , and Lock M. T. W. T. , Polypoid Arteriovenous Malformation Of The Ureter Mimicking A Fibroepithelial Polyp, A Case Report, BMC Urology. (2017) 17, no. 1, 10.1186/s12894-017-0237-z, 2-s2.0-85022182367, 28693464. PMC5504856 28693464

